# Clinical Manual of Mental Diseases

**Published:** 1898-06

**Authors:** 


**Clinical Manual of Mental Diseases.** By A. CAMPBELL CLARK, M.D. Pp. 484. London: Baillière, Tindall and Cox. 1897.

When such excellent manuals on insanity as those written by Clouston and Blandford are obtainable, we are at loss to understand why this book should have been published, and the reason for its production as set forth in the preface seems hardly sufficient. With unimportant exceptions, there is but little information which is not to be found in the manuals just mentioned, and expressed in a less pretentious manner.

It is certainly satisfactory, however, to note that Dr. Clark has considered the importance of a chapter on mental physiology, and that he has been at especial pains to elucidate the frequently obscure terms, illusion, hallucination, and delusion. It is not stated with sufficient clearness that an illusion depends upon the peculiar circumstances under which the object is presented, and not upon a faulty process of sense-perception. We do not like the term "insane illusion"; if an illusion cannot be dispelled by means sufficient for presumably normal minds, "delusion" is the proper nomenclature.

In reference to the theory of sane evolution and insane dissolution, we hold with Dr. Clark that there is no reason to expect invariably a regular unfolding in reverse order to acquisition. If, indeed, we accept a physical basis of degeneration, then it is probable that this may proceed at the same time on different planes. But the illustration used—that of the hoarding habits of the early general paralytic—does not appeal to us; the lines of commencing dissolution are too much modified by previous high developments of conduct to be compared with the acts of a boy governed by simpler motives. These habits are very likely based upon entirely different and even still complex reasonings; and do not signify a sudden retrograde plunge into early periods of development.

The lengthy *résumé* of Dr. Francis Warner's investigations into the backward and feeble-minded appears out of place in a clinical manual. This however, together with synopses of somatic insanities, undoubtedly brings the book up to date.

What we have most to complain of in the author's work is the frequently involved style of writing. In a clinical manual, when attempts at vivid description are made, terseness and lucidity should not be sacrificed. But here the meaning which the author seeks to convey is continually clouded by a choice of florid language, out of place in a work dealing with an already complex subject. By extreme tautology and a wearisome indulgence in metaphor, he succeeds often in obscuring descriptions which might otherwise have been graphic enough. Pages 19, 20 will furnish an instance of Dr. Clark's literary style—a paragraph of sixteen lines contains eleven distinct sentences and about twenty propositions; and an example of careless expression may be taken from page 190—"Homicidal impulse is often an immediate sequel of sexual disturbance—menstruation, masturbation, or sexual excess . . . the man who has done the deed seems oblivious." We imagine that the case of a man having homicidal impulse after menstruation is rare. There are, in fact, a serious number of inaccuracies and solecisms of style and grammar to be found in this work.

It is to be regretted that an antiquated system of photographic illustrations of various insanities should have been revived; they are for the most part, though well produced, not happily chosen. One of the illustrations constituting the frontispiece is in strikingly bad taste; it is surely at variance with the purpose of an intentionally serious work that one should find at the commencement a picture of an obviously sane man, wearing an impossibly false wig and beard, crouching behind an elaborately arranged bundle of straw.

